# Reliability of Two Recently Developed Procedures Assessing Biological Maturity by Ultrasound Imaging—A Pilot Study

**DOI:** 10.3390/children11030326

**Published:** 2024-03-09

**Authors:** Nicole Hutmacher, Jasmin D. Busch, Eva Rüeger, Michael Romann, Patric Eichelberger

**Affiliations:** 1 School of Health Professions, Physiotherapy, Bern University of Applied Sciences, 3008 Bern, Switzerland; 2 Department of Diagnostic, Interventional and Pediatric Radiology, Inselspital, Bern University Hospital, University of Bern, 3010 Bern, Switzerland; 3 Department of Elite Sport, Swiss Federal Institute of Sport Magglingen, 2532 Magglingen, Switzerland

**Keywords:** ultrasound, bone age, ossification ratio, biological maturity, maturity stage, youth sport, talent selection, reliability

## Abstract

During puberty, the biological maturity of children of the same chronological age differs. To generate equal opportunities for talent selection in youth sports, the athlete’s biological maturity should be considered. This is often assessed with a left hand and wrist radiography. Alternatively, ultrasound (US) could be advantageous, especially by avoiding ionizing radiation. This pilot study aimed to assess intrarater and interrater reliability of an experienced and a non-experienced examiner in an US-based examination of the knee in 20 healthy females (10–17 years). Epiphyseal closure at five anatomical landmarks was staged (stages 1–3) and its interrater and intrarater reliabilities were analyzed using Cohen’s kappa (*k*). Interrater reliability of the calculation of the ossification ratio (OssR) was analyzed using the Bland-Altman method and intraclass correlation coefficients (ICCs). Interrater reliability for the stages was almost perfect for four landmarks. Interrater reliability ranged from *k* = 0.69 to *k* = 0.90. Intrarater reliability for the stages was almost perfect for four landmarks. Intrarater reliability ranged from *k* = 0.70 to *k* = 1.0. For the OssR, ICC was 0.930 and a minimal detectable change of 0.030 was determined. To conclude, experienced and non-experienced examiners can reliably assign individuals to different ossification stages and calculate an OssR using US-based imaging of the knee.

## 1. Introduction

During childhood, but more particularly during puberty, individuals show heterogeneous growth rates, and the physiological and psychological changes that occur are rapid and distinctive [1,2]. As children approach puberty, the difference between their chronological and their biological ages may increase [3,4]. The biological age of adolescents of a similar chronological age group can vary by up to five years [5]. 

In youth sports, biological age affects physical and cognitive abilities. While chronological age influences physical fitness in preschool children [6], there is evidence that biological age has a significant effect on strength, endurance and speed in adolescents [7,8]. In youth sports, age grouping is based on chronological age using cut-off dates (1 January) [1]. Athletes who are born early in a year usually are cognitively and physically superior to those born late in the year [9,10]. The combination of the effects of chronological age with the effects of biological age can lead to substantial differences between the athletes. Cognitively and physically inferior athletes (e.g., late-born athletes and/or late developers) drop out of teams during selection processes, even though they may be equally talented [11,12].

An overrepresentation of early-born athletes in sport teams is referred to as the “relative age effect” [13]. With adequate training and sufficient time to mature physically, these talented late-developers have the chance to become more successful athletes, compared to less talented early-developers [14]. In order to actively involve late-developers in sports participation and to create fair conditions in training, competition and development, it is important to take the biological age into account in selection processes [15,16]. Knowledge of the biological age and its relation to an athlete’s chronological age helps to define the status (e.g., early-developer if biological age—chronological age ≥ 1). This status can then be used to define biological maturity. Biological maturity is about the degree of maturity, timing and tempo. Maturity status (early-, on time- and late-developer, based on biological age) refers to the state of biological maturation of an individual at the time of observation. Timing refers to the onset of maturation, while the tempo describes the rate at which it progresses. 

Biological age can be estimated using different parameters such as sexual maturity, age of teeth or bone age [17]. The determination of bone age is most commonly used to state the biological age [18], which depends on various factors such as gender, nutritional status, several hormonal, metabolic and genetic factors, the presence of acute or chronic diseases and social conditions [19]. Various imaging methods such as magnetic resonance imaging (MRI), computed tomography (CT), X-ray, DXA or US are used to measure bone age in different body parts such as the hand, wrist, clavicle, knee or the iliac crest [20]. 

X-rays of the left hand and wrist are considered the gold standard in estimating bone age [19,21]. The available analysis methods are based on the classifications according to Greulich and Pyle, Tanner-Whitehouse-2 and -3 and Gilsanz and Ratib. Furthermore, software-supported evaluations, e.g., “BoneXpert”, are becoming increasingly widespread [22].

Despite the low radiation dose in modern X-ray [23], the need for X-ray-based bone age determination without a medical indication is problematic from an ethical and legal point of view. Furthermore, in Switzerland and other Western countries, it is a legal requirement to choose the technique with the lowest radiation dose among the range of available methods (Strahlenschutzgesetz (StSG, SR 814.50)). It is therefore necessary to establish a new standardized method for radiation-free assessment of biological age. US is a radiation-free, non-invasive, cost-effective and widely available medical imaging technique and appears to be suitable from a practical, ethical and economic point of view [24]. A recent scoping review classified US assessment into four categories. 

Bone age can either be estimated in the comparative collective by comparing US images of the left hand and wrist with images of an atlas or it can be derived by calculating a maturity score. Bone maturity can be derived by staging of the ossification process or by a measurement of distances and/or calculation of a ratio [16]. 

To adjust training loads and to organize adapted competition categories, the athlete’s biological maturity should be assessed. Therefore, there is a need for a reasonable, cost-effective and practicable US-based standard method for estimation of bone maturity in youth sports [16]. In the existing literature on US-based assessment of bone maturity, there is wide diversity in the domains of application, in the methods used and in the body regions examined [16]. Despite several studies concluding good accuracy of US assessments compared to MRI or X-ray [16,25,26,27,28,29], the validity of US and its clinical utility is still under discussion [30,31]. To date, there is no literature on US-based assessment of bone maturity that considers the differences in the expertise level of the examiners [16]. The impact of the expertise level is particularly relevant in the field of sports, where an experienced physician is not always available. 

It was hypothesized that experienced and—after specific training—non-experienced examiners can reliably estimate the biological maturity of athletes using an US-based assessment. 

The aims of this pilot study therefore were to assess the interrater reliability of two examiners with different levels of expertise in an US-based assessment of five anatomical landmarks at the knee joint [32] and an US-based calculation of the ossification ratio (OssR) of the distal medial femur [24]. Furthermore, the intrarater reliability of a non-experienced examiner was assessed in an US-based examination of five anatomical landmarks at the knee joint [32].

The two US-based assessments help to estimate the biological maturity status and are therefore relevant to the determination of biological age.

## 2. Materials and Methods

### 2.1. Study Design

To serve the purpose of this pilot study, a cross-sectional study design with two measurement timepoints was chosen. The clarification of responsibility with the Ethics Committee of the Canton of Bern, Switzerland (KEK-No. Req-2022-00765) indicated that no ethical approval was required for the present study. After internal review approval, this study was conducted in accordance with the declaration of Helsinki and the European Code of Conduct for Research Integrity.

### 2.2. Participants 

Twenty healthy female Swiss handball players aged between 10 and 17 years (13.6 ± 2.3 years, mean ± sd) (Table 1) were recruited, and their personal data were coded to guarantee data protection. Adolescents were excluded if they had any form of growth disorders, musculoskeletal disorders, such as previous or current fractures in the study region, or neurological disorders. 

A written informed consent was obtained from all the participants. Additionally, a written informed consent from a legal guardian was obtained if the participant was under 13 years of age. 

### 2.3. Measurements

In November and December 2022, each participant was examined twice for epiphyseal closure within a maximum of 20 days. The examinations were performed using a written case report form. One measurement was carried out by an experienced examiner with more than 10 years of experience in the field of pediatric radiology (JB). A non-experienced examiner carried out a second measurement. The non-experienced examiner was a physical therapist who had received three days of theoretical and practical instructions in US-based imaging but who had no further experience in US imaging (NH). 

The US-based imaging of the right knee joint was performed using a commercial US imaging device (E-CUBE i7, Alpinion Medical Systems Co., Ltd., Seoul, Republic of Korea). 

A high-resolution linear transducer (Alpinion L3-12T, 3–12 MHz, 38.4 mm field of view) with a standard preset (B-mode, 10 MHz, 90dB dynamic range, 2.0 cm penetration depth) was used to ensure comparability and standardization.

**Figure 1:**
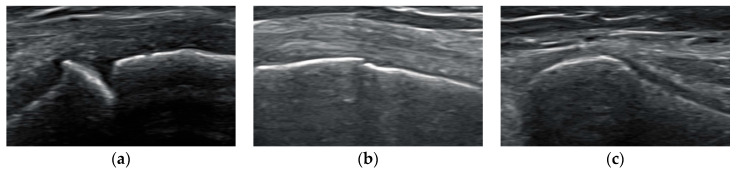
US images showing examples for stages 1–3. (**a**) Epiphyseal growth plate of the distal femur assessed from the lateral side in a 11-year-old female handball player, graded as stage 1; (**b**) epiphyseal growth plate of the proximal tibia assessed from the medial side in a 15-year-old female handball player, graded as stage 2; (**c**) epiphyseal growth plate of the proximal fibula assessed from the lateral side in a 17-year-old female handball player, graded as stage 3.

The examination was performed in a flat supine position with a distance of 50 cm between the participants’ feet. The entire growth plate in five anatomical landmarks was assessed by placing the probe longitudinally: the proximal fibular physis (Fib) from lateral, the proximal tibial physis from lateral (TL) and medial (TM) and the distal femoral physis from lateral (FL) and medial (FM) [32]. On every landmark, an image was taken of the location where the growth plate was the widest (Figure 1).

**Figure 2:**
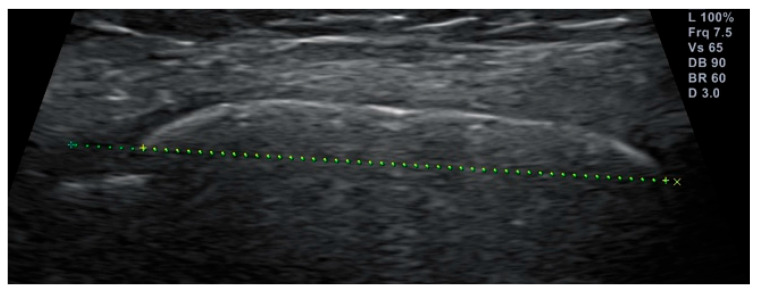
US image showing the measurements to calculate the ossification ratio (OssR) of a 10-year-old female handball player’s medial femur condyle. The green dotted line represents the maximum epiphyseal diameter. The yellow dotted line represents the diameter of the ossification center.

Afterwards, a virtual convex image was taken from the ossification center and epiphysis of the medial distal femoral epicondyle, placing the probe alongside the medial collateral ligament [24]. Virtual convex imaging allows for an expanded field of view, using a linear probe (Figure 2). An image guide of the US-based examinations can be found in the Supplementary Materials.

### 2.4. Grading

The two examiners (JB and NH) independently and blindly evaluated the six US images from the participants they examined within two months after examination. To determine not only the interrater reliability but also the intrarater reliability, NH reevaluated the images of Fib, TL, TM, FL and FM after seven days. 

The grade of epiphyseal closure in Fib, TL, TM, FL and FM was determined using a three-point scale [32] (Table 2). The three stages are illustrated in Figure 1.

The convex image of the ossification center and epiphysis of the medial distal femoral epicondyle was used to calculate the OssR as described by Wan et al. [24], where the diameter of the ossification center is divided by the maximum epiphyseal diameter. First, the maximum epiphyseal diameter was measured along the long axis of the femur, starting from the junction between the epiphysis and diaphysis and terminating at the distal end of the epiphysis. Afterwards, the projection of the ossification center on this line was measured (Figure 2). 

### 2.5. Statistical Analysis 

Statistical analysis was performed using R software (version 4.4.2.). Descriptive statistics were calculated for the demographic characteristics of the sample. Statistical significance was set at *p* < 0.05. 

Descriptive statistics and an unweighted Cohen’s kappa (*k*) [33] using psych::cohen.kappa (v2.2.9) were calculated to examine the interrater and intrarater reliability of the stages of Fib, TL, FL, TM and FM. The reliability was considered poor for *k* < 0.00, slight for 0.00 ≤ *k* ≤ 0.20, fair for 0.21≤ *k* ≤ 0.40, moderate for 0.41 ≤ *k* ≤ 0.60, substantial for 0.61 ≤ *k* ≤ 0.80 and almost perfect for *k* > 0.80 [34]. A Pearson’s Chi-squared test was then performed using stats::chisq.test (v4.2.2) to analyze the dependence of the agreement rate between the examiners from the stage. Further, Odds ratios (ORs) were calculated to measure the association between the stage of epiphyseal closure and the probability of a disagreement between the two examiners. 

For statistical analyses of the OssR and of the absolute diameters of the ossification center and the epiphysis that were measured to calculate the OssR, mean and standard deviation (SD) were calculated. The absolute interrater reliability was analyzed using the Bland–Altman method calculating the systematic error (bias) and limits of agreement (LoA) with 95% confidence intervals (CIs) [35,36,37,38] using blandr::bland.altman (v0.5.1). The absolute and the relative minimal detectable change (MDC) were calculated. The minimal detectable change (MDC) was calculated as 1.96 × SDdifferences (standard deviation of the differences). The MDC indicates the smallest amount of change that goes beyond measurement error and is used to differentiate between true changes and changes caused by errors. Furthermore, intraclass correlation coefficients ICC(A,1) values [39], representing the relative reliability, were calculated, using irr::icc (v0.84.1).

## 3. Results

### 3.1. Stages 

To calculate the interrater reliability, the ratings from the two examiners were used (JB, NH). To state the intrarater reliability, NH rated her images twice (NH and NH2). Stage 1 was observed 35 times by JB, 32 times by NH and 32 times by NH2. Stage 2 was observed 62 times by JB, 64 times by NH and 65 times by NH2. Stage 3 was observed three times by JB, four times by NH and three times by NH2.

#### 3.1.1. Interrater Reliability

The interrater reliability between the two examiners (JB und NH) was analyzed for Fib, TL, FL, TM and FM by calculating Cohen’s kappa (*k*). The interrater reliability was almost perfect for each of these five locations except for FM, where the interrater reliability was found to be substantial (Table 3). 

#### 3.1.2. Dependence of the Interrater Reliability from the Stage

**Figure 3:**
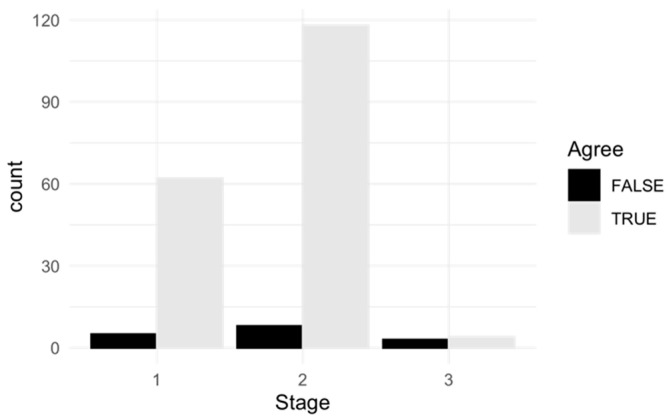
Number of agreement (true) and disagreement (false) cases between the two examiners JB and NH per stage (1–3).

The dependency of the level of agreement between the two raters from the stage of epiphyseal closure was analyzed by the absolute and the relative frequencies of agreement (true) and disagreement (false) per stage (Table 4, Figure 3). In a Pearson’s Chi-squared test, the p-value of 0.002 revealed that the agreement between JB and NH depended on the stage. It was 9.3-times more probable (OR = 9.3) that the raters disagreed in stage 3 compared to that in stage 1. It was less probable that the examiners disagreed in stage 2 compared to that in stage 1 (OR = 0.8).

#### 3.1.3. Intrarater Reliability

The intrarater reliability was almost perfect for each of these five locations except for TM, where the intrarater reliability was found to be *k* = 0.70 and therefore was substantial (Table 3). 

### 3.2. Ossification Ratio

**Figure 4:**
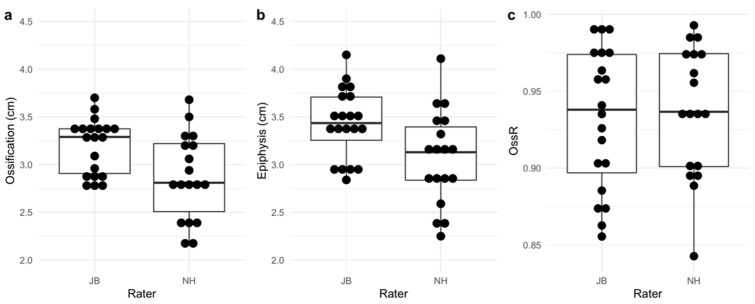
Diameter of the ossification centers (**a**); diameter of the epiphyses (**b**); and ossification ratios (OssRs) (**c**) calculated by the two examiners JB and NH.

Descriptive analyses of the OssR showed small differences between the examiners JB and NH. The mean OssR measured by JB was 0.933 (SD = 0.046). NH measured a mean OssR of 0.937 (SD = 0.042) (Figure 4c). Due to inferior image quality, NH could not calculate the OssR in two subjects. For completeness and to allow a more accurate comparison, the two diameters (ossification center and epiphysis) that were used for calculation of the OssR were also tested for interrater reliability. For the ossification center, JB found a mean of 3.19 cm (SD = 0.286) and NH found a mean of 2.87 cm (SD = 0.444) (Figure 4a). The means for the epiphysis diameter were 3.43 cm (SD = 0.365) as measured by JB and 3.07 cm (SD = 0.495) as measured by NH (Figure 4b). 

**Figure 5:**
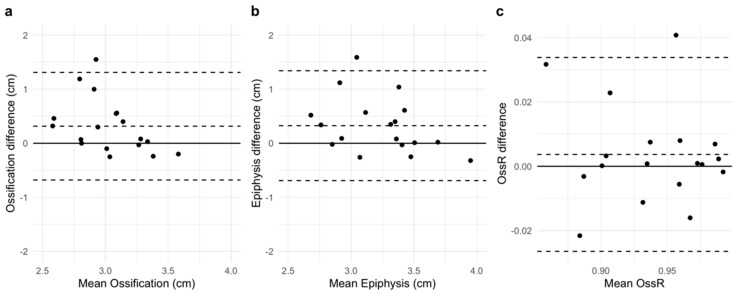
BlandAltman plots for a comparison of (**a**) the diameter of the ossification centers; (**b**) the epiphyseal diameter and (**c**) the ossification ratio (OssR) measured by JB and NH. The dashed lines in the middle represent the bias (mean difference); the outer dashed lines represent the upper and the lower limits of agreement.

For absolute interrater reliability, the Bland–Altman analysis for the diameter of the ossification center showed an MDC of 0.995 with a 95% CI of [0.787, 1.204]. The bias was 0.316 (SD = 0.508) with a 95% CI of [0.064, 0.569] (Figure 5a). The ICC(A,1) value for relative reliability was found to be 0.047 with a 95% CI of [−0.272, 0.429]. The Bland–Altman analysis for the diameter of the epiphysis showed an MDC of 1.016 cm with a 95% CI of [0.803, 1.229]. The bias was 0.326 (SD = 0.519) with a 95% CI of [0.068, 0.583] (Figure 5b). The ICC(A,1) value for relative reliability was found to be 0.205 with a 95% CI of [−0.162, 0.569]. The Bland-Altman analysis for the OssR showed an MDC of 0.030 with a 95% CI of [0.024, 0.036]. The bias was 0.004 (SD = 0.015) with a 95% CI of [−0.004, 0.011] (Figure 5c). Moreover, the ICC(A,1) value for relative reliability was found to be 0.930 with a 95% CI of [0.828, 0.973]. The values for the MDC, the bias and the ICC(A,1) are presented in Table 5.

## 4. Discussion

The aims of this pilot study were to evaluate the interrater and intrarater reliabilities of an US-based examination of five anatomical landmarks (Fib, TL, FL, TM, FM) of the right knee joint [32] and to evaluate the interrater reliability of an US-based calculation of the OssR of the distal medial femur [24] to state biological maturity. It was hypothesized that experienced and—after specific training—non-experienced examiners can reliably estimate the biological maturity of athletes using an US-based assessment. Results of this pilot study show good reliability. The results of this study are discussed in the following chapters. The overall high values for the relative inter- and intrarater reliabilities show that there is a high potential for the use of US-based imaging, for example, in the field of sports where an experienced physician is not always available and where X-ray-based bone age determination is problematic from an ethical and legal point of view as there is no medical indication. It gets even more interesting when considering the mobility of US devices, the reduction in the expenditure of time compared to X-ray or MRI-based examinations, the lower costs and the high correlation between MRI and US staging of bone maturity [32]. An overrepresentation of early developers and relative age effects are common in competitive sports [40,41]. Bio-banding is a concept where players are organized in groups based on their biological age rather than their chronological age for training and competition to ensure equal opportunities [42]. If ultrasound-based estimation of biological maturity proves to be reliable and valid, it could endorse such systems to maintain fairness among young athletes in competitions and selection processes.

### 4.1. Interrater Reliability (Maturity Stages)

The interrater reliability of the US-based examination of the maturation stage of Fib, TL, FL, TM and FM was almost perfect for Fib, TL, FL and TM. For FM, the interrater reliability was rated as substantial. However, when analyzing the 95% CIs of the *k*-values of Fib, TL, FL, TM, it was found that reliability can range from moderate to almost perfect. The 95% CI of [0.37, 1.00] of the *k*-value of FM indicates that the interrater reliability can be fair to almost perfect. 

To the best of the authors’ knowledge, there are only two studies evaluating the interrater reliability of an US-based staging procedure at the knee joint to evaluate biological maturity. In both studies, relative reliability was assessed using Cohen’s kappa (*k*) statistics and obtained *k*-values similar to those of the present study. The two studies obtained *k*-values ranging from 0.813 to 0.952 [32,43]. In contrast to the present study, the investigators in the studies described were all experienced, with at least two years of practice in the study by Windschall et al. [43] and five years in the study by Herrmann et al. [32]. Other studies analyzing the interrater reliability of US-based assessments between an experienced and a non-experienced rater found similarly high values for relative reliability, ranging from ICC 0.84 to ICC 0.987 [44,45]. The two studies examined muscle thickness and grey scale. However, Herrmann et al. [32] and Windschall et al. [43] recorded slightly higher *k*-values overall. These results imply that US-based assessments of bone maturity—especially on the medial distal femur—are challenging for non-experienced examiners. Another reason for slightly higher *k*-values might be a variation in image generation and the grading system. Herrmann et al. [32] used a cine-loop analysis of the same anatomical landmarks that were graded using the same grading system as in the present study. Windschall et al. [43] used a four-degree scale to classify the suprapatellar (longitudinal probe positioning, knee joint in 30° flexion) and the lateral (transversal probe positioning, knee joint in a neutral position) region of the knee. Furthermore, Herrmann et al. [32] found lower values for interrater agreement for stage 2 than for stage 1 and 3. In the present study, a dependence of the relative reliability on the stage was also observed. Calculation of the odds showed that agreement was highest for stage 2. Due to the age of the subjects, stage 2 was found most frequently. There was a similar number of disagreements (ranging from 3 to 8) between JB and NH in all stages. This could have led to the higher percentage of agreement in stage 2. 

Each of the three stages was observed in the sample, with stage 3 being least frequent. This can be explained by the mean age of 13.6 years (SD = 2.257) in the sample. In a previous radiographic study, the earliest complete growth plate closure in knee joints of females was found at 13.82 years of age [46]. Moreover, a recently published MRI study found that only 19.4% of 14-year-old females had complete growth plate closure in the proximal tibia [47]. 

### 4.2. Intrarater Reliability (Maturity Stages)

The intrarater reliability of the US-based examination of the maturity stage of Fib, TL, FL, TM and FM was found to be almost perfect in Fib, TL, FL and FM. For TM, the intrarater reliability was found to be substantial. However, when analyzing the 95% CIs of the *k*-values of Fib, TL, FL and FM, it was found that the reliability can range from substantial to almost perfect. The 95% CI of [0.31, 1.00] of the *k*-value of TM indicates that the intrarater reliability can be fair to almost perfect. The confidence intervals are wide as the number of persons examined is small. 

Windschall et al. [43] found *k*-values for the intrarater reliability of 0.88. To the best of the authors’ knowledge, there is no other study that has analyzed the intrarater reliability of an US- based assessment of bone maturity at the knee joint. In a previous review, 20 studies were found that assessed biological maturity with some type of staging procedure at body regions other than the knee [16]. Furthermore, five of these studies assessed intrarater reliability and found *k*-values ranging from 0.862 to 0.971 [48,49,50], ICC values of 0.836 and 0.967 [51] and an intraview reliability of 96% [28].

### 4.3. Interrater Reliability (OssR)

The relative interrater reliability of the US-based examination of the OssR of the medial distal femur proved to be excellent with an ICC(A,1) of 0.930. This is similar to the ICC value of 0.93 determined by Wan et al. [24]. The comparability of the results should be questioned as Wan et al. [24] measured the OssR not only on the medial femur, but also on many other body regions. They observed a high correlation of 0.91 (Pearson’s r) between bone age and the OssR of the medial femur in girls, which was the highest correlation for single bones they found. On this basis, the present study focused on this body region. For absolute reliability, the MDC was found to be 0.030 with a 95% CI of [0.024, 0.036]. Despite the high absolute interrater reliability of the calculated OssR, analyzing the measured distances for the ossification center and epiphysis itself, the interrater reliability was extremely low, as the relative MDCs were 33% and 31%, respectively. The values for the bias obtained from the Bland–Altman analysis showed that the non-experienced examiner measured shorter distances for the ossification centers and the epiphyses than the experienced examiner (Figure 5, Table 5). Furthermore, the boxplots for the ossification center and the epiphysis show a higher variability in the measurements of NH (Figure 4). Between the examiners, the values for the median differed more for the measurements of the ossification center than for those of the epiphyses. The values for the bias showed that the systematic error was significant. It is hypothesized that it is more difficult for non-experienced examiners to scan the structures of the medial distal femur, as the interrater reliability was also lowest for FM. Furthermore, measuring the OssR appears to be more challenging than simply staging growth plates, as the non-experienced examiner was unable to calculate the OssR in two cases due to insufficient image quality.

### 4.4. Limitations

The present study has several limitations. It is a pilot study with a small number of subjects with a high degree of homogeneity—all subjects were healthy female handball players with the same ethnic background. The results are therefore not generally transferable. Research on a larger and more heterogeneous group therefore should be planned in the future. The two examiners were not tested for agreement with raters of the same expertise level. It is therefore not known how representative they are of the groups of experienced and non-experienced examiners, respectively. The interrater reliability of the image generation and the process of image reading was not analyzed individually. Therefore, it is not possible to state whether it was rather the process of image generation or the process of image analysis (e.g., staging, measuring the OssR) that differed between the two examiners with different expertise levels. To better differentiate, future research should calculate the interrater reliability between the two examiners, as they rated the same images. Furthermore, intrarater reliability was only tested for the non-experienced examiner and only for the process of image reading. It is possible that the values for the intrarater reliability would have been different if image generation had been taken into account. 

## 5. Conclusions

To conclude, the findings of this study indicate that experienced and—after specific training—non-experienced examiners as well can reliably assign individuals to different ossification stages using an ultrasound-imaging technique at the right knee. Calculating the ossification ratio on the distal medial femur is reliable as well, even though the absolute distances measured in order to calculate the ossification ratio differed when measured by examiners with different expertise levels. The overall high inter- and intrarater reliabilities show the high potential for using this ultrasound-based imaging for the purpose of youth sport, where an experienced physician is not always available and where X-ray-based bone age determination is problematic from an ethical and legal point of view as there is no medical indication. A practicable assessment of biological age is important to actively involve late-developers in sports participation and to create fair conditions in training, competition and development. 

Because of several limitations and the study design of the present study, the results need to be interpreted with caution. Further research with larger numbers and a higher heterogeneity of subjects (e.g., athletes with different sports background) are needed to further evaluate reliability and therefore the possible implementation of ultrasound in the field of sport. Furthermore, the validity, e.g., the accordance between bone maturity or bone age determined by US and by an X-ray or MRI image, needs to be explored in the future.

## Figures and Tables

**Table 1:** Characteristics of the 20 participants.

	Mean	SD	Median	Min	Max
Age (in years)	13.6	2.3	13.5	10	17
Body height (in cm)	161.5	10.1	163.5	142	175
Body weight (in kg)	52.6	11.3	57.5	32	68

**Table 2:** US-based grading of the growth plate closure for Fib, TL, TM, FL and FM as proposed by Herrmann et al. [32].

	Definition of Stages 1–3
Stage 1	The growth plate is open and there is a large gap (2–3 mm) between epiphysis and metaphysis. From the outer cortex into the physis, there is a right-angle step-off (metaphyseal zone of calcification) (Figure 1a).
Stage 2	The growth plate has a small diameter. There is only a shallow notch between epiphysis and metaphysis (Figure 1b).
Stage 3	The growth plate is closed. No gap can be seen between epiphysis and metaphysis (Figure 1c).

**Table 3:** Interrater and intrarater reliability: Cohen’s kappa and 95% CI for the five locations Fib, TL, FL, TM and FM of the right knee.

	Cohen’s Kappa [95% CI]	
	Interrater Reliability (JB, NH)	Intrarater Reliability (NH, NH2)
Fibula (Fib)	0.82 [0.60, 1.00]	1.00 [1.00, 1.00]
Lateral tibia (TL)	0.90 [0.70, 1.00]	1.00 [1.00, 1.00]
Lateral femur (FL)	0.89 [0.67, 1.00]	1.00 [1.00, 1.00]
Medial tibia (TM)	0.86 [0.60, 1.00]	0.70 [0.31, 1.00]
Medial femur (FM)	0.69 [0.37, 1.00]	0.88 [0.66, 1.00]

**Table 4:** Absolute and relative frequency of agreement (true) and disagreement (false) between the two examiners JB and NH per stage (1–3).

	Agreement		Disagreement		Row Total	
	Absolute	Relative	Absolute	Relative	Absolute	Relative
Stage 1	62	31%	5	2.5%	67	33.5%
Stage 2	118	59%	8	4%	126	63%
Stage 3	4	2%	3	1.5%	7	3.5%
Column total	184	92%	16	8%	200	100%

**Table 5:** Overview of interrater agreement and reliability values for the ossification ratio and the absolute diameter of the ossification center and the epiphysis.

	Bias [95% CI] in cm	Absolute MDC [95% CI] in cm	Relative MDC [95% CI] %	ICC(A,1) [95% CI]
OssR	0.004	0.030	3.225	0.930
	[−0.004, 0.011]	[0.024, 0.036]	[2.550, 3.901]	[0.828, 0.973]
Diameter ossification center	0.316	0.995	32.718	0.047
	[0.064, 0.569]	[0.787, 1.204]	[25.865, 39.571]	[−0.272, 0.429]
Diameter epiphysis	0.326	1.016	31.155	0.205
	[0.068, 0.583]	[0.803, 1.229]	[24.629, 37.680]	[−0.162, 0.569]

## Data Availability

The data are available on OLOS (https://doi.org/10.34914/olos:qguqt2lgrbartphgkck5eypfrq). The data was accessed on 7 March 2024.

## Supplementary Materials

The following supporting information can be downloaded at: https://www.mdpi.com/article/10.3390/children11030326/s1, Image guide of US-based examinations.
