# Supplementary material for: Reliability of Two Recently Developed Procedures Assessing Biological Maturity by Ultrasound Imaging—A Pilot Study
Source: Children (Basel). 2024 Mar 9;11(3):326. doi: 10.3390/children11030326 (PMC10968870; doi:10.3390/children11030326)
Supplement: Supplementary file 1 [file children-11-00326-s001.zip › children-2890406-supplementary.pdf]

Image Guide

Reliability of two recently developed procedures assessing biological maturity by ultrasound imaging

Position 1	Epiphysis **Fibula** 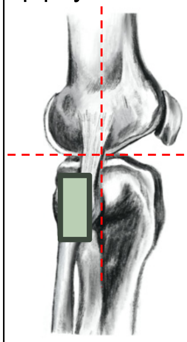
Position 2	Epiphysis **Tibia lateral** 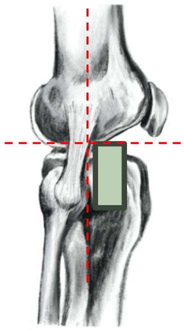
Position 3	Epiphys **Femur lateral** 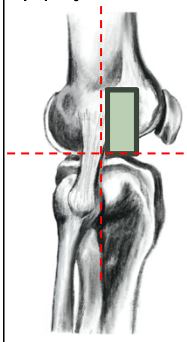

Position 4	Epiphysis **Tibia medial** 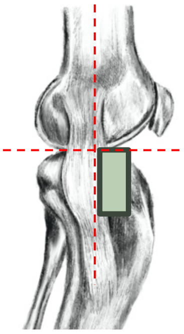
Position 5	Epiphysis **Femur medial** 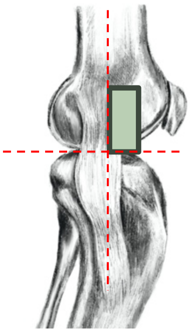
Position 6	Ossification ratio **Femur medial** 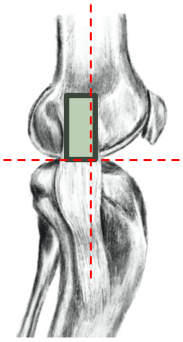

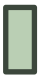 = placement of the US-probe

References:

1) Own images
